# Antioxidant-rich leaf extract of *Barringtonia racemosa* significantly alters the *in vitro* expression of genes encoding enzymes that are involved in methylglyoxal degradation III

**DOI:** 10.7717/peerj.2379

**Published:** 2016-08-25

**Authors:** Kin Weng Kong, Azlina Abdul Aziz, Nurhanani Razali, Norhaniza Aminuddin, Sarni Mat Junit

**Affiliations:** 1 Department of Molecular Medicine, Faculty of Medicine, University of Malaya, Kuala Lumpur, Malaysia; 2 Institute of Biological Sciences, Faculty of Science, University of Malaya, Kuala Lumpur, Malaysia

**Keywords:** *Barringtonia racemosa*, Leaf extract, Gene expression, Microarray analysis, Ingenuity Pathways Analysis, Methylglyoxal degradation III

## Abstract

**Background:**

*Barringtonia racemosa* is a medicinal plant belonging to the *Lecythidaceae* family. The water extract of *B. racemosa* leaf (BLE) has been shown to be rich in polyphenols. Despite the diverse medicinal properties of *B. racemosa*, information on its major biological effects and the underlying molecular mechanisms are still lacking.

**Methods:**

In this study, the effect of the antioxidant-rich BLE on gene expression in HepG2 cells was investigated using microarray analysis in order to shed more light on the molecular mechanism associated with the medicinal properties of the plant.

**Results:**

Microarray analysis showed that a total of 138 genes were significantly altered in response to BLE treatment (*p* < 0.05) with a fold change difference of at least 1.5. *SERPINE1* was the most significantly up-regulated gene at 2.8-fold while *HAMP* was the most significantly down-regulated gene at 6.5-fold. Ingenuity Pathways Analysis (IPA) revealed that “Cancer, cell death and survival, cellular movement” was the top network affected by the BLE with a score of 44. The top five canonical pathways associated with BLE were Methylglyoxal Degradation III followed by VDR/RXR activation, TR/RXR activation, PXR/RXR activation and gluconeogenesis. The expression of genes that encode for enzymes involved in methylglyoxal degradation (*ADH4*, *AKR1B10* and *AKR1C2*) and glycolytic process (*ENO3, ALDOC* and *SLC2A1*) was significantly regulated. Owing to the Warburg effect, aerobic glycolysis in cancer cells may increase the level of methylglyoxal, a cytotoxic compound.

**Conclusions:**

BLE has the potential to be developed into a novel chemopreventive agent provided that the cytotoxic effects related to methylglyoxal accumulation are minimized in normal cells that rely on aerobic glycolysis for energy supply.

## Background

Plants have been widely used for thousands of years as food and medicine. However, much ethno-medico botanical knowledge is unrecorded and the underlying molecular mechanisms are yet to be identified. *Barringtonia racemosa* (L.) Spreng belongs to the *Lecythidaceae* family and grows wild in the tropics. *B. racemosa* is a species native to the Philippines but its habitat covers a broad range of area including Eastern Africa, Madagascar, India and other Indian Ocean islands, Asia and Southeast Asia, Northern Australia, Melanesia, Micronesia and Polynesia. In Malaysia, the shoots of *B. racemosa* are usually consumed as salad. There are diverse traditional uses of the leaves including for reducing high blood pressure, to relieve cough and as a depurative (Ong & Nordiana, 1999). Among the Malaysian aboriginal ethnics, the pounded leaves, roots and bark are applied externally to reduce skin itchiness (Hanum & Hamzah, 1999). Scientifically, the leaf extract of *B. racemosa* has been reported to show anti-tumor activities in B-lymphoblastoid Raji cells (Murakami et al., 2000) and anti-proliferative effect in cervical carcinoma, HeLa cell line (Mackeen et al., 1997). Other than that, the methanolic extract of the seeds showed anti-tumor activities in mice challenged with Dalton’s Lymphoma Ascitic cells (Thomas et al., 2002). The methanolic extract of the fruits exhibited anti-proliferative effect in breast cancer MCF-7 cells (Amran et al., 2016). Rutin, isolated from the fruits of *B. racemosa* showed anti-proliferative activities towards leukemic cells like JURKAT, MOLT 3, REH and K562, and its cytotoxic effect on the normal human peripheral blood mononuclear cell was not detected (Samanta et al., 2010).

Despite the diverse medicinal properties of *B. racemosa*, information on its major biological effects and precise molecular mechanisms involved are still lacking. Biological functions of a living cell are highly dependent on changes in gene expression, as a response to alteration in extracellular conditions. As the shoots of *B. racemosa* are commonly consumed, information particularly on the effect of edible leaf portion of *B. racemosa* on gene expression would be beneficial, to support its medicinal claims. Recent advances in molecular technology including cDNA microarray have provided invaluable tool to further understand the therapeutic mechanisms of medicinal plants. Microarray analysis was able to provide a comprehensive molecular signature in cells in response to plant extracts such as *Tamarindus indica* (*T. indica*) fruit pulp (Razali, Aziz & Junit, 2010), *T. indica* leaves (Razali et al., 2015) and *Anacardium occidentale* shoots (Khaleghi et al., 2011).

The leaf water extract of *B. racemosa* (BLE) was reported to possess antioxidant properties that correlated well to its polyphenolic and ascorbic acid content (Kong et al., 2012). Chromatographic analysis revealed that gallic acid, protocatechuic acid, ellagic acid, quercetin and kaempferol are the major polyphenolic compounds found in the leaf (Kong et al., 2014).

HepG2 cells have been widely used as an *in vitro* model in nutrigenomics studies since they retain many of the specialized functions of normal human hepatocytes (Mersch-Sundermann et al., 2004) and showed closest similarity in terms of signaling network patterns with those observed in primary hepatocytes (Saez-Rodriguez et al., 2011). Hence, in this study, the direct effects of the antioxidant-rich BLE on gene expression in HepG2 cells that can be linked to the medicinal properties of the plant were investigated.

## Methods

### Sample preparation

*Barringtonia racemosa* shoots were collected in February 2011 from Kedah, Malaysia. The voucher specimen (KLU48175) of the sample was deposited in the Herbarium of Rimba Ilmu, University of Malaya. The leaf portion was separated from the shoots and subjected to lyophilization. The lyophilized sample was ground and sieved via a 1 mm mesh before extracted in water. The extract was centrifuged and the supernatant was then collected and filtered using Whatman No. 4 before lyophilization. The lyophilized leaf extract of *B. racemosa* (BLE) was re-dissolved in deionized water and filtered via 0.22 µm membrane filter before being stored at −20 °C. A stock solution of the sample was further diluted using the Dulbecco’s modified Eagle’s media (DMEM) medium (Lonza Walkersville, MD, USA).

### Cell culture

Human hepatoma HepG2 cell line was obtained from the American Type Culture Collection (ATCC) (Manassas, VA, USA). Cells were cultured in DMEM with 2.0 g/l sodium bicarbonate, antibiotics (100 units of penicillin/ml and 100 µg of streptomycin/ml) and 10% fetal bovine serum (FBS). Cells were maintained in a humidified atmosphere of 5% CO_2_ at 37 °C.

### Supplementation of HepG2 cells with BLE

HepG2 cells were seeded in T-75 cm^2^ culture flasks at 1.2 × 10^6^ cells per flask. Cells were stabilized for 24 h followed by treatment with a final concentration of 50 µg/ml of BLE for a further 24 h. A control group was prepared by replacing the same volume of BLE with DMEM. Three biological replicates were done for each treatment. After the 24 h treatment, cells were washed thrice with PBS prior to trypsinization and centrifugation at 16,200×*g* for 5 min. Cell pellet was collected and used for total cellular RNA (tcRNA) extraction.

### Preparation of total cellular RNA (tcRNA)

TcRNA of the untreated (control) and BLE-treated HepG2 cells was extracted and purified using an RNeasy Mini kit (Qiagen, Germany) according to the provided procedures. The cells were lysed using 350 µl of a guanidine thiocyanate containing buffer (RLT buffer) mixed with beta-mercaptoethanol (*β*-ME). Following this, lysed cells were homogenized in a QIAshredder and the homogenate was treated with DNase I. TcRNA was then collected following centrifugation-aided elution using RNeasy spin column. The quality of the tcRNA was determined by the ratio of the optical density at 260 nm and 280 nm measured using a spectrophotometer. A ratio of the A260/A280 between 1.8 and 2.1 indicates that the tcRNA is of good quality. Using a denaturing gel electrophoresis, the integrity of the tcRNA was evaluated, based on the presence of two distinct, clear bands that correspond to the ribosomal 28S and 18S. The 28S rRNA band should be approximately twice as intense as that of the 18S. The yield of the tcRNA was quantitated spectrophotometrically at 260 nm. An absorbance of 1 unit corresponds to 40 µg/ml of tcRNA.

### Preparation of sense strand DNA for microarray analysis

Gene expression analysis was performed using Affymetrix Human Gene 1.0 S.T (sense target) array based on the conventional Affymetrix eukaryotic RNA labelling protocol. The extracted tcRNA (200 ng) was reverse transcribed into single-stranded sense strand DNA or complementary DNA (cDNA) in two cycles via the whole transcript (WT) cDNA synthesis, amplification kit and purification module. Then, the cDNA was subjected to fragmentation using a mixture of uracil-DNA glycosylase (UDG) and apurinic/apyrimidinic endonuclease 1 (APE1). The fragmented cDNA was subsequently end-labeled by terminal deoxynucleotidyl transferase (TdT) via a terminal transferase reaction incorporating biotinylated dideoxynucleotides. The fragmented and biotin-labeled amplified cDNA (2.5 µg) was then hybridized to the Affymetrix Human Gene 1.0 S.T array at 45 °C for 18 h in a Gene Chip Hybridization Oven 640. After hybridization, the array was stained and washed in the Affymetrix GeneChip Fluidics Station 450 under a standard procedure. The stained array was then scanned at 532 nm using an Affymetrix GeneChip Scanner 3000 7G. The CEL file generated using the Affymetrix Gene-Chip^®^ Operating Software (GCOS) was used for data analysis. Data were subjected to normalization, background correction and data summarization using Affymetrix Expression Console Software prior to further analysis.

### Microarray data normalization and analysis

The CEL files generated by GCOS were exported to Partek Genomic Suite Software and the gene list generated was then normalized by filtering out the probeset IDs that did not have any annotation in the Partek Genomic Suite Software, using the NetAffx Analysis Centre Software. The gene sets were then subjected to a one-way analysis of variance (ANOVA) in the Partek Genomic Suite Software to determine significantly (*p* < 0.05) expressed sets of genes. Significantly expressed genes were then re-filtered to include only those with fold change difference of equal to or greater than 1.5. Microarray data were further analyzed using the Gene Ontology Enrichment tool in the Partek Genomic Suite Software. Genesis Expression Similarity Investigation Suite software was used to compare the genes regulation in different treated groups to show the reproducibility of the current dataset (Sturn, Quackenbush & Trajanoski, 2002).

### Biological network analyses of significantly regulated genes using Ingenuity Pathways Analysis (IPA) software

The transcriptomic data were further analyzed using the Ingenuity Pathways Analysis (IPA) software (Ingenuity^®^ Systems, www.ingenuity.com) to predict networks and canonical pathways that are linked to the significantly expressed genes. Details of the significantly altered genes, their quantitative expression values (fold change difference of at least 1.5) and *p*-values (*p* < 0.05) were imported into the IPA software. Each identifier was mapped to its corresponding protein object and was overlaid onto a global molecular network developed from information contained in the Ingenuity Knowledge Base (Ingenuity^®^ System, www.ingenuity.com). Network of genes were then algorithmically generated based on their connectivity. Right-tailed Fischer’s exact test was used to calculate a *p*-value indicating the probability that each biological function assigned to the network and canonical pathway is due to chance alone.

### Validation of the microarray data using real-time polymerase chain reaction

Microarray data were validated by quantitative real time-PCR (qRT-PCR) using a StepOne™ Real-Time PCR System. The primer pairs for the selected up-regulated and down-regulated genes as well as a housekeeping gene (*GADPH*) are listed in File S1. tcRNA was first converted to cDNA using a high capacity RNA to cDNA master mix (Applied Biosystem, USA) prior to PCR amplification. PCR amplification was done in 0.2 ml MicroAmp^®^ Optical 8-tube strips in a final volume of 20 µl containing a mixture of cDNA (10 ng), reverse and forward primers (200 nM), pre-prepared Fast SYBR^®^ Green master mix containing SYBR^®^ Green 1 dye, AmpliTaq^®^ Fast DNA Polymerase (ultra pure), Uracil-DNA glycosylase (UDG), ROX™ dye passive reference, dNTPs and the optimized buffer components. PCR parameters consisted of 40 cycles of amplification with initial denaturation at 95 °C for 15 s and annealing elongation at 60 °C for 60 s. The PCR mixture was initially held for 10 min at 95 °C prior to the PCR cycles. The comparative C_*T*_ (threshold cycle) method (ΔΔC_*T*_) was chosen for the relative quantification of gene expression. mRNA levels of the selected genes were normalized against that of *GADPH*.

## Results

### Gene expression profiling in BLE-treated HepG2 cells

**Figure 1:**
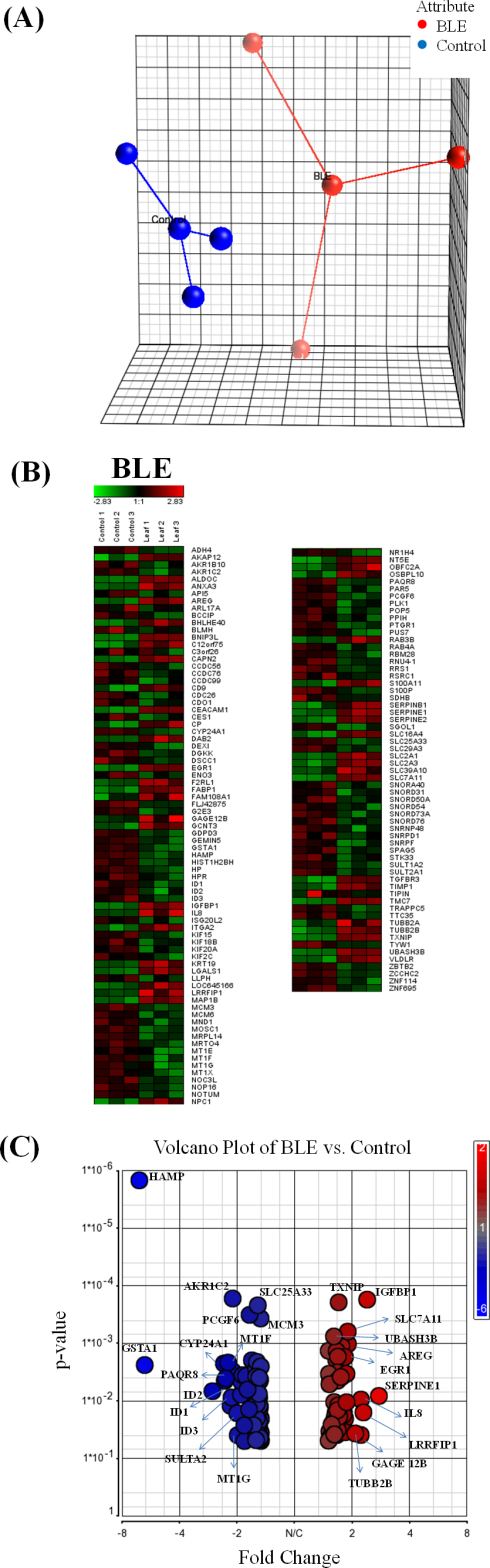
(A) Principal component analysis (PCA) plot generated from biological replicates of HepG2 cells in control (blue) and BLE (red) treatment groups; and (B) hierarchical cluster analysis showing genes that were significantly expressed (*p* < 0.05) by at least 1.5-fold in HepG2 cells treated with BLE as compared to control. Green indicates down-regulation. Red indicates up-regulation. BLE, leaf water extract of *B. racemosa*. (C) Volcano plot of genes that were significantly regulated (*p* < 0.05) by at least 1.5-fold in HepG2 cells treated with BLE. Red indicates up-regulation and blue indicates down-regulation.

The extracted tcRNA from control and BLE-treated HepG2 cells for cDNA microarray analysis was of good quality, indicated by the A260/A280 ratio of above 1.8 and of good integrity determined by the presence of two distinct bands of the ribosomal 28S and 18S with the former approximately twice as intense as the latter (File S2). Principle component analysis (PCA) plot of microarray data from each array for the different treatment groups are shown in Fig. 1A. Biological replicates (*n* = 3) of the control and BLE-treated groups are represented in blue and red, respectively. Sample outliers were not detected in all of the studied groups.

Hierarchical clustering analysis was performed to indicate reproducibility of the experiments. Genes that were significantly regulated (*p* < 0.05) with a fold change difference greater than or equal to 1.5 fold in HepG2 cells treated with BLE are shown in Fig. 1B. Down-regulated genes are indicated in green while up-regulated genes are presented in red. A similar pattern of color representing the regulation of the genes was observed within the biological replicates of each of the studied group indicating reproducibility of the experiments. A volcano plot (Fig. 1C) shows the distribution of the significantly regulated genes in response to BLE of which the up-regulated genes are represented in red while the down-regulated genes are in blue.

When the hybridization signals of the BLE-treated cells were compared to those of the control group, a total of 138 genes were significantly altered (*p* < 0.05) by at least 1.5-fold in the HepG2 cells. Twenty significantly altered genes with fold change of at least 2 are tabulated in Table 1. *SERPINE1* was the top significantly up-regulated gene at 2.8-fold while *HAMP* was the top significantly down-regulated gene at 6.5-fold in response to the antioxidant-rich BLE. A full list of the significantly regulated genes in HepG2 cells in response to BLE treatment is provided in File S3.

**Table 1:** Genes significantly regulated by BLE, at 2 < fold change < − 2.

No.	GenBank ID	Gene symbol	Encoded protein	Fold-change
1.	NM_000602	*SERPINE1*	Serpin peptidase inhibitor, clade E (nexin, plasminogen activator inhibitor type 1)	2.8
2.	NM_000596	*IGFBP1*	Insulin-like growth factor binding protein 1	2.4
3.	NM_001137550	*LRRFIP1*	Leucine rich repeat (in FLII) interacting protein 1	2.3
4.	NM_001127345	*GAGE12B*	G antigen 12B	2.2
5.	NM_000584	*IL8*	Interleukin 8	2.2
6.	NM_178012	*TUBB2B*	Tubulin, beta 2B	2.1
7.	NM_001657	*AREG*	Amphiregulin	2.0
8.	NM_005950	*MT1G*	Metallothionein 1G	−2.0
9.	NM_003167	*SULT2A1*	Sulfotransferase family, cytosolic, 2A, dehydroepiandrosterone	−2.0
10.	NM_000670	*ADH4*	Alcohol dehydrogenase 4 (class II), pi polypeptide	−2.1
11.	NM_001354	*AKR1C2*	Aldo-keto reductase family 1, member C2 (dihydrodiol dehydrogenase)	−2.1
12.	NM_002167	*ID3*	Inhibitor of DNA binding 3, dominant negative helix-loop-helix protein	−2.1
13.	NM_020299	*AKR1B10*	Aldo-keto reductase family 1, member B10 (aldose reductase)	−2.2
14.	NM_005949	*MT1F*	Metallothionein 1F	−2.2
15.	NM_181353	*ID1*	Inhibitor of DNA binding 1, dominant negative helix-loop-helix protein	−2.3
16.	NM_133367	*PAQR8*	Progestin and adipoQ receptor family member VIII	−2.3
17.	NM_000782	*CYP24A1*	Cytochrome P450, family 24, subfamily A, polypeptide 1	−2.4
18.	NM_002166	*ID2*	Inhibitor of DNA binding 2, dominant negative helix-loop-helix protein	−2.7
19.	NM_145740	*GSTA1*	Glutathione S-transferase alpha 1	−6.1
20.	NM_021175	*HAMP*	Hepcidin antimicrobial peptide	−6.5

### Network and pathway analyses of significantly regulated genes using IPA software

The top five biological networks associated with genes that were significantly altered by the antioxidant-rich BLE are listed in Table 2. “Cancer, Cell Death and Survival, Cellular Movement” is the top putative network associated to BLE treatment, linking 24 out of 35 significantly regulated genes (File S4) with other interactomes, with a score of 44. A score of 2 or higher indicates at least a 99% confidence that a network is not being generated by random chance. A higher score represents a better confidence level. Most of the genes were linked to digestive organ tumor but eight were specifically linked to colorectal cancer namely *AREG/AREGB*, *CD9, CEACAM1*, *IL8,* and *SLC2A3* (or *GLUT3*), *MCM3*, *MT1E* and *MT1F*. Other biological networks affected by the BLE, in descending scores, were “Cancer, Cardiac Dilation, Cardiovascular System Development and Function” (32), “Cell Cycle, Connective Tissue Development and Function, Cellular” (27), “Metabolic Disease, Drug Metabolism, Endocrine System Development” (25), and “Behavior, Cell-To-Cell Signaling and Interaction, Drug Metabolism” (21).

**Table 2:** IPA analyses of the top networks and predicted canonical pathways associated to the significantly altered expression genes in HepG2 cells in response to BLE treatment.

Top network	Score
1. Cancer, Cell Death and Survival, Cellular Movement	44
2. Cancer, Cardiac Dilation, Cardiovascular System Development and Function	32
3. Cell Cycle, Connective Tissue Development and Function, Cellular Development	27
4. Metabolic Disease, Drug Metabolism, Endocrine System Development and Function	25
5. Behavior, Cell-To-Cell Signaling and Interaction, Drug Metabolism	21

Top canonical pathway	*P* value	Up-regulated gene	Down-regulated gene
1. Methylglyoxal degradation III	7.40E–05	–	*ADH4, AKR1B10, AKR1C2*
2. VDR/RXR activation	2.33E–03	*IGFBP1, SERPINB1*	*CYP24A1, SULT2A1*
3. TR/RXR activation	3.18E–03	*RAB3B, SLC2A1*	*AKR1C2, HP*
4. PXR/RXR activation	1.21E–02	*IGFBP1*	*GSTA1, SULT2A1*
5. Gluconeogenesis I	1.26E–02	*ALDOC*	*ENO3*

**Notes.**

The analyses were based on the significantly regulated (*p* < 0.05) genes of BLE treatment (138 genes). BLE, leaf water extract of *B. racemosa*; VDR, vitamin D receptor; RXR, retinoic X receptor; TR, thyroid hormone receptor; PXR, pregnane X receptor.

**Figure 2:**
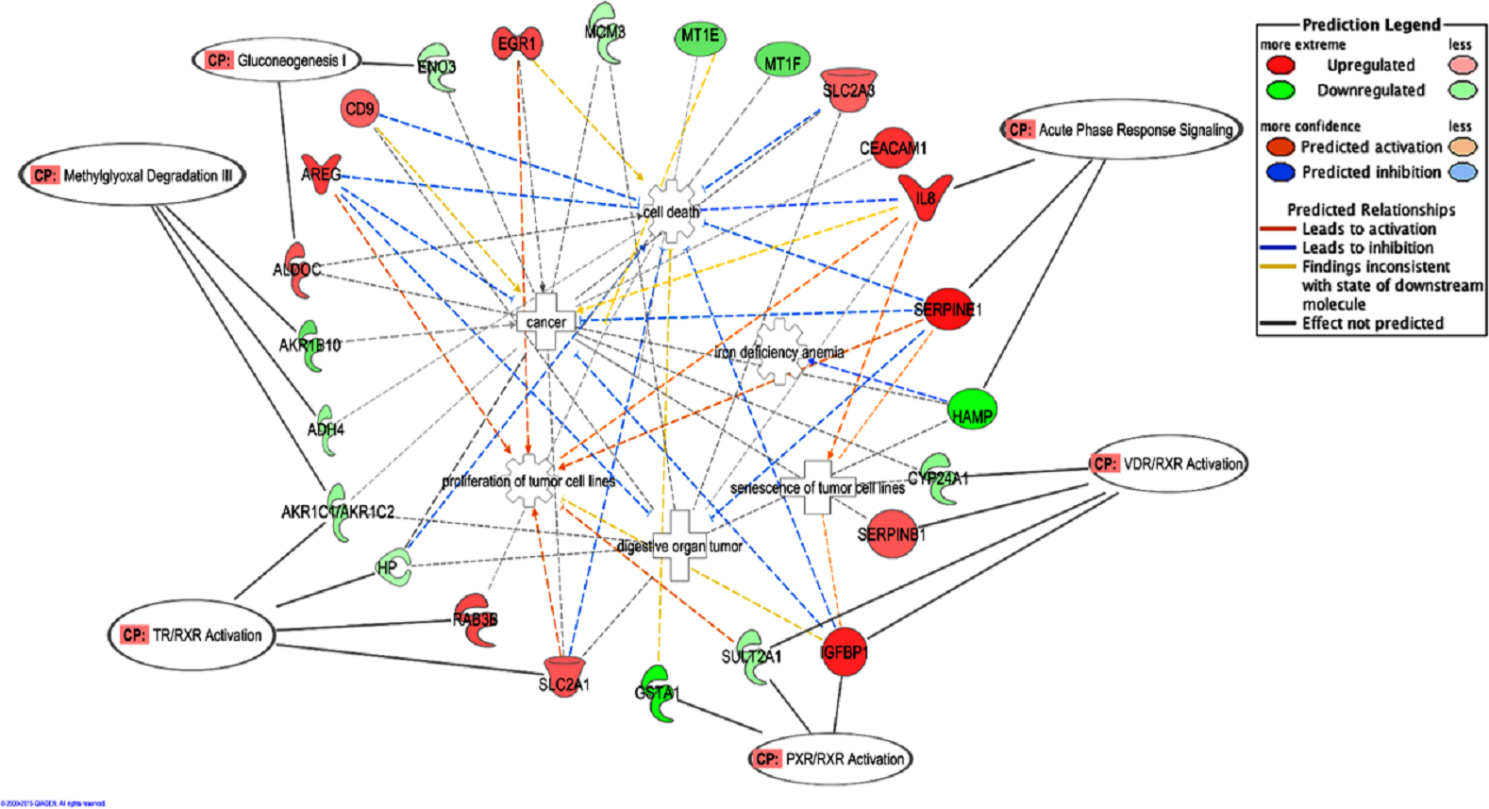
A graphical representation of the molecular relationships between the expression of genes that were significantly affected by the BLE treatment associated to the top canonical pathways as well as genes related to colorectal cancer that found in “Cancer, Cell Death and Survival, Cellular Movement” network of BLE vs control treatment. The network is displayed graphically as nodes (genes) and edges (the biological relationships between the nodes). Nodes in red indicate up-regulated genes while those in green represent down-regulated genes. Various shapes of the nodes represent the functional class of the proteins. Edges are displayed with various colors that describe the nature of the relationship between the nodes. Names of genes corresponding to the abbreviations are as follows: ADH4, Alcohol dehydrogenase 4 (class II), pi polypeptide; ALDOC, Aldolase C, fructose-bisphosphate; AKR1B10, aldo-keto reductase family 1, member B10; AKR1C2, Aldo-keto reductase family 1, member C2 (dihydrodiol dehydrogenase); AREG/AREGB, amphiregulin; CD9, tetraspanin CD9; CEACAM1, carcinoembryonic antigen-related cell adhesion molecule 1; CYP24A1, cytochrome P450, family 24, subfamily A, polypeptide 1; EGR1, Early growth response 1; ENO3, Enolase 3 (beta, muscle); GSTA1, Glutathione S-transferase alpha 1; HAMP, Hepcidin antimicrobial peptide; HP, Haptoglobin; IGFBP1, insulin-like growth factor binding protein 1; IL8, interleukin 8; MCM3, minichromosome maintenance complex component 3; MT1E, metallothionein 1E; MT1F, metallothionein 1F; RAB3B, RAB3B, member RAS oncogene family; SERPINB1, Serpin peptidase inhibitor, clade B (ovalbumin), member 1; SERPINE1, Serpin peptidase inhibitor, clade E (Nexin, Plasminogen activator inhibitor, type 1), member 1; SLC2A1, Solute carrier family 2 (facilitated glucose transporter), member 1; SLC2A3, Solute carrier family 2 (facilitated glucose transporter), member 3; SULT2A1, Sulfotransferase family, cytosolic, 2A, dehydroepiandrosterone.

The top five canonical pathways affected by BLE were Methylglyoxal degradation III (*p* < 7.40 × 10^−05^) followed by VDR/RXR activation (*p* < 2.33 × 10^−03^), TR/RXR activation (*p* < 3.18 × 10^−03^), PXR/RXR activation (*p* < 1.21 × 10^−02^) and gluconeogenesis (*p* < 1.26 × 10^−02^) (Table 2). Figure 2 depicts the predicted molecular relationships between the significantly regulated genes that were associated to the top canonical pathways. *ADH4* and *AKR1B10* were the two genes associated with the Methylglyoxal Degradation III while *AKR1C2* were linked to both Methylglyoxal Degradation III and TR/RXR activation. *IGFBP1* and *SULT2A1* were both linked to the second and fourth canonical pathways, VDR/RXR activation and PXR/RXR activation respectively, while *SERPINB1* and *CYP24A1* were only associated with the VDR/RXR activation. *SLC2A1* and *HP* were only linked to the TR/RXR activation while *GSTA1* was only linked to the PXR/RXR activation (Table 2). *SERPINE*1 and *HAMP* are listed under the canonical pathway, “Acute Phase Response Signaling”. IPA analysis suggests that BLE affects the Acute Phase Response Signaling by altering the expression of *IL8* and *SERPINE1*; converging mainly on senescence of tumor cell lines, while *HAMP* functions in the inhibition of iron deficiency anemia (Fig. 2).

### Validation of the microarray data

**Figure 3:**
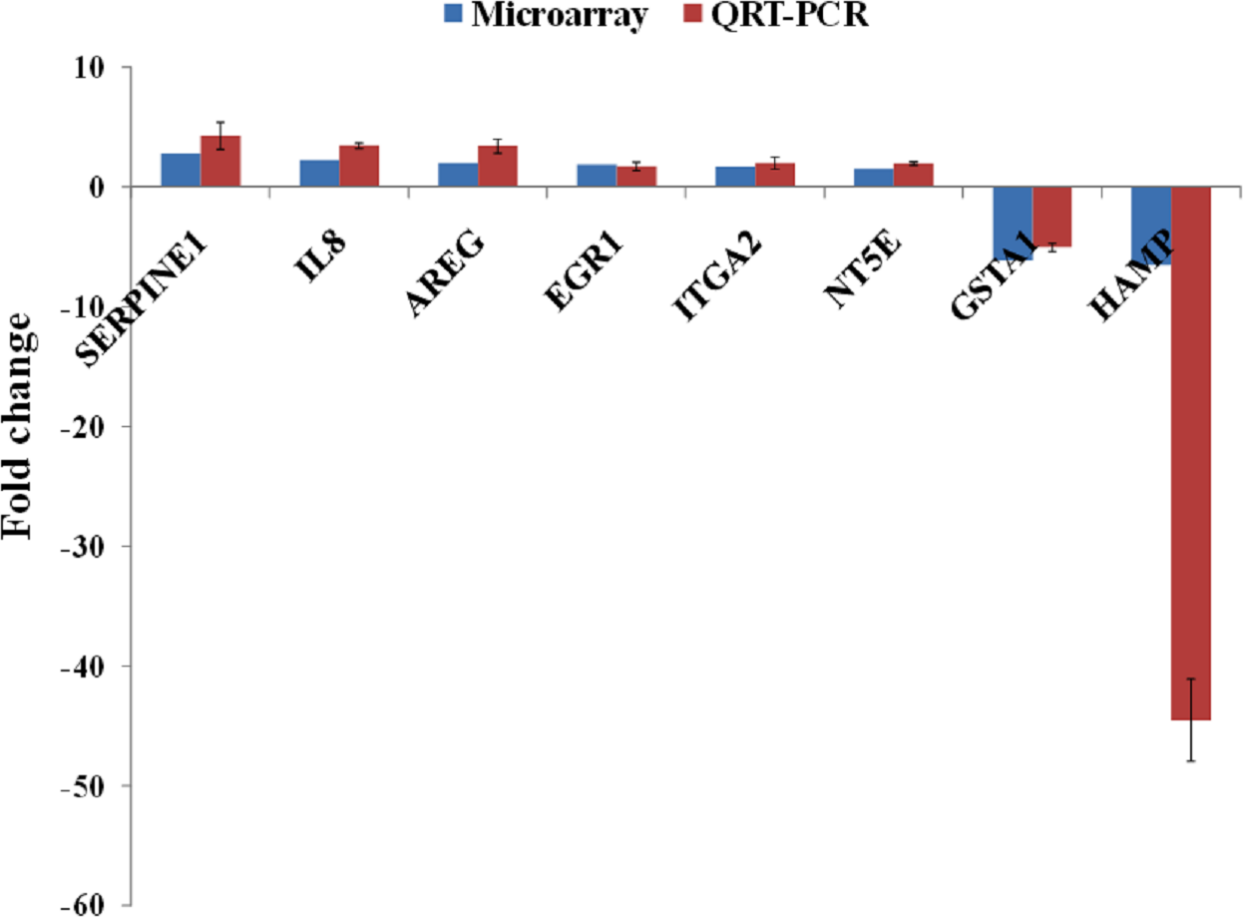
qRT-PCR validation of microarray gene expression data for BLE treatment. BLE, leaf water extract of *B. racemosa*.

A group of significantly up- and down-regulated genes was chosen from Table 1, and their expressions were quantitated using quantitative real time-PCR (qRT-PCR). As shown in Fig. 3, the expression of *SERPINE1, IL8, AREG, EGR1, ITGA2* and *NT5E* were up-regulated while, *HAMP* and *GSTA1* were down-regulated in BLE-treated HepG2 cells. Identical patterns of gene expression changes were observed as in the microarray data.

## Discussion

Our group has recently reported that BLE is rich in antioxidants and polyphenols including gallic acid, protocatechuic acid, ellagic acid, quercetin and kaempferol (Kong et al., 2014). In addition to polyphenols, ascorbic acid was also detected in the BLE (Kong et al., 2012). The human plasma levels of polyphenolic compounds are roughly between 0.001–6 µM or 15 µM, when integrated with proteins (Boulton, Walle & Walle, 1998; Spencer, 2008) while ascorbic acid content is commonly less than 70 µM (Lykkesfeldt & Poulsen, 2010). Fifty µg/ml of BLE used in this study contained gallic acid (6.17 µM), ellagic acid (2.29 µM), protocatechuic acid (1.77 µM), quercetin (1.47 µM), kaempferol (0.74 µM) and ascorbic acid (25.23 µM), which were within the levels normally detected in plasma (Kong et al., 2012; Kong et al., 2014). Antioxidant-rich plant extracts have been shown to be able to directly influence the expression of genes in HepG2 cells (Razali, Aziz & Junit, 2010) and hamsters (Lim et al., 2013) which was corroborated by alteration in protein abundance (Chong et al., 2013). In this study, the antioxidant-rich BLE was also able to significantly alter the expression of a total of 138 genes in the same *in vitro* model. From our experience, 24 h exposure to plant extract is sufficient for cells to show response at the gene expression level. This period of exposure was also adopted by other researchers working in similar areas (Luo et al., 2008; Qin et al., 2012; Zhang et al., 2011). *SERPINE1* was the top significantly up-regulated gene at 2.8-fold while *HAMP* was the most significantly down-regulated gene at 6.5-fold, in response to the antioxidant-rich BLE. *SERPINE1* encodes PAI-1, a single chain glycoprotein and a class E member of the serine protease inhibitor (SERPIN). PAI-1 functions as a central regulator of various injury-initiated cellular processes including cell migration, growth, senescence and survival in several organ sites (Simone & Higgins, 2015). Cellular senescence can be regarded as the physiological endstate of the proliferative capacity of cells. The concept of pro-senescence therapy has emerged over the past few years as a novel therapeutic approach to treat cancers (Mileo & Miccadei, 2015). Emerging evidence has demonstrated that therapy-induced senescence is a critical mechanism through which many anticancer drugs inhibit tumor progression (Ewald et al., 2010). PAI-1 is a critical downstream target of tumor suppressor p53 in the induction of replicative senescence. p53 controls growth factor-dependent proliferation of cells by up-regulating PAI-1, leading to down-regulation of PI(3)K-PKB signaling and nuclear exclusion of cyclin D1 (Kortlever, Higgins & Bernards, 2006). PAI-1 inhibits the activity of the secreted protease urokinase type plasminogen activator (uPA) by forming a stable complex. uPA expression can cause growth of cells to progress through activating a mitogenic signalling cascade of PI(3)K-PKB and activating cyclin D1 thus increasing the bioavailability of growth factors (Chandrasekar et al., 2003). A previous study reported that oncogenic cells become unresponsive to mitogenic signaling by antagonizing uPA through p53mediated up-regulation of PAI1 (Kortlever & Bernards, 2006). Additionally, several secreted proteins including insulin-like growth factor binding proteins (IGFBPs), cytokines such as IL6, and ligands of the chemokine receptor, CXCR2 have been shown to mediate or reinforce cells senescence (Acosta et al., 2008). It is interesting to show that the expression of *IGFBP1* and *IL8*, a chemokine activated by *CXCR2*, were also up-regulated in this present study, thus it is speculated that the BLE may have an anticancer property that may involve the mediation of cancer cells senescence. Quercetin contained in BLE extract might contribute to this anticancer property as it has been reported to induce the expression of genes including *IL8* which mediate tumor cells senescence (Chuang et al., 2010).

*HAMP* encodes hepcidin, a key iron regulatory hormone secreted by the liver to control systemic iron homeostasis (Ganz & Nemeth, 2012). Hepcidin and its receptor, ferroportin, inhibits iron entry into the plasma compartment, dietary absorption in the duodenum, the release of recycled iron from macrophages and the release of stored iron from hepatocytes (Ganz & Nemeth, 2012). Both iron deficiency and iron excess can cause cellular and organ dysfunction. Hepcidin synthesis by hepatocytes is suppressed by erythropoietic activity while systemic inflammatory diseases increase hepcidin synthesis through IL-6 and other mediators, leading to anemia of inflammation or anemia of chronic diseases. Hepcidin antagonists should be useful for treatment of iron-restrictive anemia. A novel synthetic compound, K7174 significantly down-regulated *HAMP* expression in HepG2 cells thus, it may be a potential therapeutic option to treat anemia of chronic disease (Fujiwara et al., 2013). Hence, BLE might work in a similar mechanism as K-7174 in HepG2 cells. Quercetin, one of the bioactive compounds found in BLE (Kong et al., 2014), could be responsible for the regulatory effect on *HAMP* gene expression as similar effect was observed in rats fed with quercetin (Lesjak et al., 2014) which subsequently affect iron absorption.

When the 138 genes were further analyzed using knowledge-based Ingenuity Pathways analyses software, the majority of the regulated genes were linked to “Cancer, Cell Death and Survival, Cellular Movement”. “Colorectal cancer” was identified as the more specific cancer that was linked to the BLE-treated group. From the top canonical pathways analyses, BLE is predicted to exert its anti-cancer effect through the methylglyoxal degradation pathway III. Cancer cells are known to have a higher rate of energy metabolism (Furuta et al., 2010). The best characterized metabolic phenotype observed in tumor cells is the Warburg effect, which is a shift from ATP generation through oxidative phosphorylation to ATP generation through glycolysis, even under normal oxygen concentration (Warburg, 1956). As a result, unlike most normal cells, many tumor cells derive a substantial amount of their energy from glycolysis, converting most incoming glucose to lactate rather than metabolizing it in the mitochondria through oxidative phosphorylation (Furuta et al., 2010; Warburg, 1956).

Methylglyoxal is an elimination product/byproduct of triose phosphate isomerization in glycolysis (Pun & Murphy, 2012) and from the metabolism of threonine and acetone (Ahmed et al., 1997). In cell culture models, methylglyoxal has been shown to be cytotoxic as it acts as a precursor for glycation leading to the formation of advanced glycation end products (AGEs) (Pun & Murphy, 2012). However, methylglyoxal is also a regulator of cell growth, which could inhibit proliferation of cancer cells (Együd & Szent-Györgyi, 1968) by preventing glycolysis and mitochondrial respiration (Biswas et al., 1997; Ghosh et al., 2006; Ray & Ray, 1998). A previous study has shown that methylglyoxal selectively inhibited both mitochondrial respiration and glycolysis in human leukaemic leucocyte cells whereas the respiration in normal cells remained unaffected under identical conditions of incubation (Biswas et al., 1997). As a consequence of inhibition of both mitochondrial respiration and glycolysis, ATP levels in these malignant cells have been found to be critically reduced, rendering the cells non-viable (Halder, Ray & Ray, 1993). Methylglyoxal has also been found to inactivate glyceraldehyde-3-phosphate dehydrogenase (GA3PD), a key enzyme of the glycolytic pathway, and this inactivation is responsible to a significant extent for the inhibition of glycolysis of tumor cells by methylglyoxal (Halder, Ray & Ray, 1993). These findings paved the way for anticancer drug development using methylglyoxal as a key component (Talukdar et al., 2008). Data on the toxicity effects of methylglyoxal in animal studies are however inconsistent (Kalapos, 1999). Absence of detrimental effect in normal mice, rats, rabbits and dogs treated with different doses of methylglyoxal via oral, subcutaneous and intravenous routes has been reported (Ghosh et al., 2006).

Co-administration of methylglyoxal and ascorbic acid in mice augmented the anticancer effect without any recorded toxicity (Ghosh et al., 2006). Interestingly, BLE is an extract that is not only rich in polyphenols but also in ascorbic acid (Kong et al., 2012), which may provide the same augmentation effect. The augmentation of methylglyoxal effect by ascorbic acid was reported to be resulted from the formation of protein aldehyde adducts in cancer cells but detailed mechanism remains unknown (Ray et al., 1991). Structural chemistry research indicates that there is a coupling interaction between methylglyoxal and ascorbic acid through a hydrogen bonding system (Banerjee et al., 2004). Thus, it was presumed that a slight structural change of methylglyoxal caused by ascorbic acid probably prevent the former from enzymatic degradation and at the same time increase its specificity to the malignant cancer cells (Banerjee et al., 2004).

One of the major obstacles with using methylglyoxal as an anticancer agent is that methylglyoxal, being a normal metabolite, is rapidly degraded by various enzymes present in the body (Pal et al., 2015; Ray & Ray, 1998). Naturally, there are several pathways to degrade methylglyoxal including methylglyoxal degradation I to VIII pathways. However, in *Homo sapiens*, methylglyoxal is metabolized by methylglyoxal degradations I, III and IV pathways. Methylglyoxal degradation I involves the glyoxalase system, methylglyoxal degradation III is based on aldo-keto reductase and aldose reductase, while methylglyoxal degradation IV is associated to the activities of aldehyde dehydrogenase and alcohol dehydrogenase (Kalapos, 1999; Romero et al., 2005). Biologically, enzymes such as aldose reductase, aldo-keto reductase, aldehyde dehydrogenase and glyoxalase are needed to degrade the intracellular methylglyoxal by transforming it into alcohol, pyruvate, glycolate or glycolic acid (Pun & Murphy, 2012). In methylglyoxal degradation pathway III, methylglyoxal is reduced to acetol by several methylglyoxal reductases and acetol is subsequently converted to L-1,2-propanediol by NADPH-dependent aldose reductase and aldo-keto reductase subfamily. L-1,2-propanediol is then converted by alcohol dehydrogenase to form an alcohol, (*S*)-propane-1,2-diol, before being transported out of cells (Romero et al., 2005). In this study, BLE significantly down-regulated the expression of *ADH4*, *AKR1B10* and *AKR1C2* genes that encode for alcohol dehydrogenase 4 (class II), aldo-keto reductase family 1, member B10 (aldose reductase) and aldo-keto reductase family I, member C2 (dihydrodiol dehydrogenase) respectively, of the methylglyoxal degradation III pathway. These enzymes are involved specifically in the degradation of methylglyoxal. A number of previous studies have been done to prevent this *in vivo* degradation, to increase methylglyoxal efficiency by prolonging its circulation time in blood, thus making it more competent as an anticancer drug (Chakrabarti et al., 2014; Sinha et al., 2006). In fact, a group of researchers has developed a nanofabrication of methylglyoxal with chitosan biopolymer, a nanoparticulate drug delivery system to protect it from degradation. This protection enhanced the efficacy of methylglyoxal as an anticancer drug in the tested Ehrlich ascites carcinoma, human breast cancer and lung epithelial adenocarcinoma cell lines that is augmented by creatine and ascorbic acid (Pal et al., 2015).

The suppression of *AKR1B10* in this study further strengthens the anticancer properties of BLE. Apart from its physiological functions, a growing body of evidence indicates an important role of AKR1B10 in the development of various tumorous diseases. It participates in several physiological reactions such as the reduction of retinal to retinol (Gallego et al., 2006), thus balancing the homeostasis of retinoic acid, a signaling molecule that modulates cell proliferation and differentiation and the reduction of isoprenyl aldehydes namely farnesal and geranylgeranial to metabolites involved in protein prenylation, a process considered to be a crucial event in carcinogenesis (Chung et al., 2012). AKR1B10 also catalyzes the reduction of different types of carbonyl compounds, including products of oxidative stress or drugs (Zhong et al., 2011), as well as participates in the activation of procarcinogens (Quinn, Harvey & Penning, 2008). In comparison with normal tissues, AKR1B10 is considerably overexpressed in several types of cancer such as hepatocellular (Tsuzura et al., 2014), lung, breast, gastric and pancreatic (Yao et al., 2014). Due to the potentiation of these activities, the enzyme is believed to be implicated in the development of these diseases.

Quercetin was reported to inhibit the activity of AKR1B10 in catalyzing the reduction of retinal to retinol and the reduction of isoprenyl aldehydes (Zemanova et al., 2015). Hence, in addition to ascorbic acid, quercetin might potentiate the anti-tumor properties of BLE by down-regulating the expression of *ADH4*, *AKR1B10* and *AKR1C2*, thus preventing the degradation of methylglyoxal.

**Figure 4:**
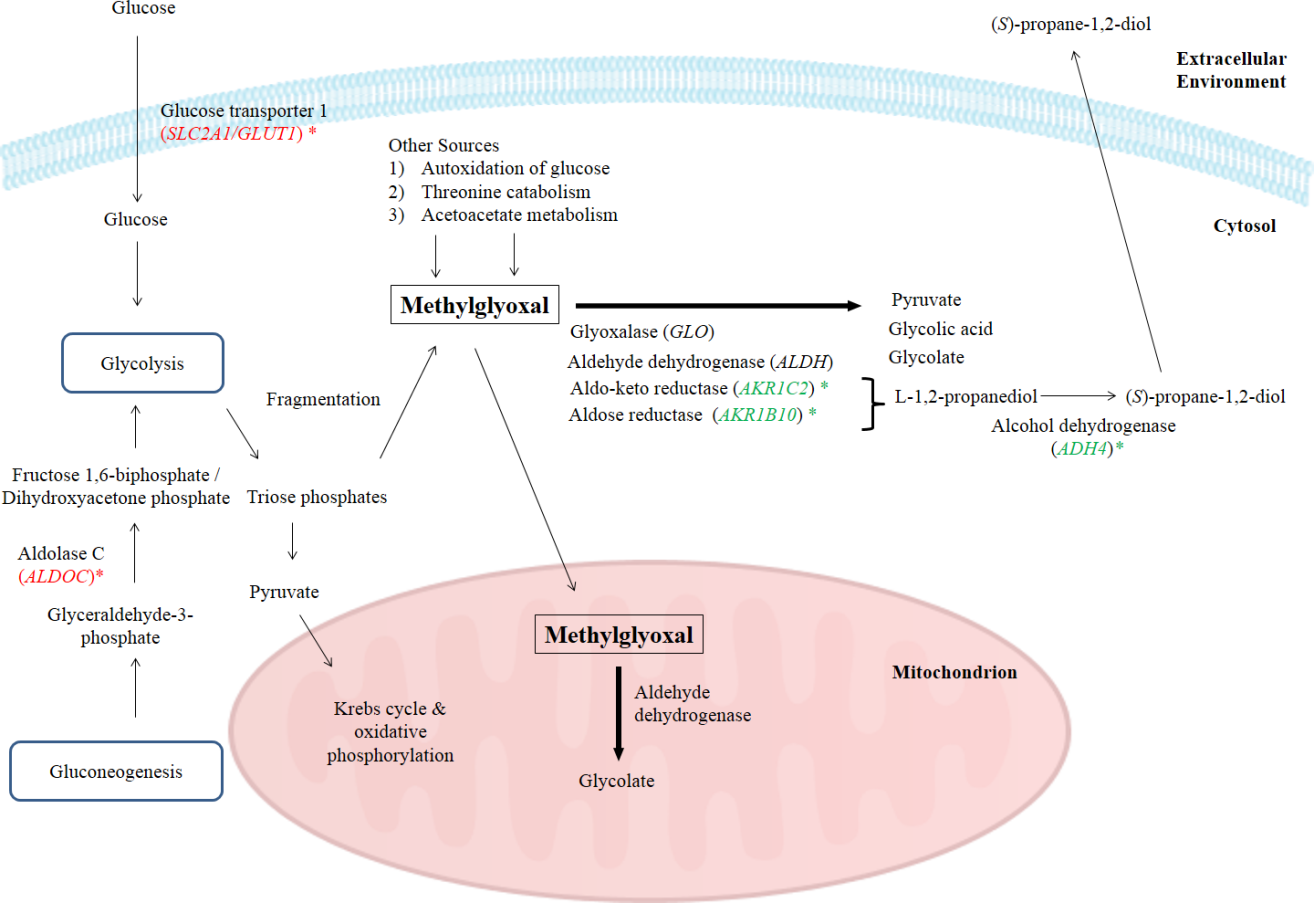
A graphical representation to illustrate how BLE may affect cellular metabolism of methylglyoxal. Genes that were down-regulated by BLE are indicated as ( 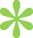) while those that were up-regulated are shown in ( 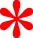). The source of information is from this study and Kalapos (1999); Pun & Murphy (2012) and Romero et al. (2005).

In this study, the suppression of *ADH4*, *AKR1B10* and *AKR1C2* genes and the subsequent encoded proteins may help to increase the intracellular methylglyoxal level which can subsequently lead to apoptosis in cancer cells. Furthermore, over-expression of enzymes related to methylglyoxal detoxification especially glyoxalase was widely reported in human malignancies (Thornalley & Rabbani, 2011). Hence, lack of methylglyoxal as a cytotoxic agent in malignant cells will promote the growth and metastasis of cancer tumor (Thornalley & Rabbani, 2011). Figure 4 illustrates the possible mechanisms related to the enhancement of methylglyoxal by proteins in which their genes were regulated by BLE treatment.

As high energy metabolism in cancer cells will lead to increased need in energy supply, hence this has prompted them to rely on other energy sources. This probably explains the up-regulation of *ALDOC*, a gene that codes for Aldolase C (ALDOC). ALDOC is involved in both glycolysis and gluconeogenesis that may further enhance the production of methylglyoxal (Fig. 4).

*SLC2A1* encodes the solute carrier family 2 facilitated glucose transporter 1 or glucose transporter 1 (GLUT1). GLUT1 is a transporter that controls glucose uptake across the cell membrane in all cell types (Hruz & Mueckler, 2001). In this study, *SLC2A1* was up-regulated by BLE treatment. The expression of this transporter for glucose uptake is well-correlated to the Warburg effect in cancerous cells and it has a great influence on the tumorigenic features (Ganapathy, Thangaraju & Prasad, 2009). It is hypothesized that the cytotoxic effect of methylglyoxal in cancer cells will be promoted by a synergistic effect of the up-regulation of *SLC2A1* gene and the down-regulation of genes encoding enzymes that are involved in the degradation of methylglyoxal. Furthermore, previous study reported that GLUT1 can function as a receptor for the uptake of ascorbic acid (Montel-Hagen et al., 2008). This may enhance the uptake of ascorbic acid as well as augment the effect of methylglyoxal in cancer cells.

Other than the gene encoding enzymes in methylglyoxal pathway III, the expression of *ENO3* which code for beta-enolase that is involved in glycolysis and gluconeogenesis, was significantly down-regulated. In gluconeogenesis, this enzyme catalyzes the hydration of phosphoenolpyruvate (PEP) to phospho-D-glycerate (PGA) (Trojanowicz et al., 2009). Thus, down-regulation of *ENO3* gene expression and subsequently the encoded protein may prevent the growth of cancer cells. The possibility of BLE as an anticancer agent in epithelial cancers is hypothesised due to the augmentation effect on methylglyoxal via regulation of genes in methyglyoxal degradation pathway and part of the genes regulated in the top network. A detailed study is suggested to further elucidate the effect of BLE on colorectal cancer.

The progression of many metabolic diseases is fundamentally regulated at the transcriptional level by a family of nuclear receptors as ligand-activated transcription factors, which detect and respond to metabolic changes. Their role in maintaining metabolic homeostasis makes nuclear receptors an important pharmaceutical and dietary target (Germain et al., 2006). In this study, BLE treatment also induced the activation of type II nuclear receptors complex including VDR/RXR, TR/RXR and PXR/RXR. Type II nuclear receptor includes vitamin D receptor (VDR), the thyroid receptor (TR), retinoic acid receptor (RAR), retinoid X receptor (RXR), pregnane X receptor (PXR) and peroxisome proliferator-activated receptors (PPARs). A particularly intriguing feature of type II receptors is that many of them function as heterodimers with RXR (Mangelsdorf & Evans, 1995). In this regard, RXR appears to have a central role in the actions of those type II receptors (Mangelsdorf & Evans, 1995).

The VDR–RXR complex binds to the vitamin D response elements (VDREs) through the DNA-binding domain in the promoters of target genes. Alterations in VDR expression, and in the synthesis (25-hydroxylase and 1*α*-hydroxylase) (Zinser & Welsh, 2004) and catabolism (24-hydroxylase) of vitamin D metabolites are involved in the growth regulation of tumors (Deeb, Trump & Johnson, 2007). Studies reported that the major vitamin D catabolizing enzyme, CYP24A1 (24-hydroxylase), is often amplified and overexpressed in tumor cells (Anderson et al., 2006). Hence, agents that inhibit this enzyme can potentiate anti-tumor effects.

The TR/RXR complex attracts a large number of proteins, which engage the RNA polymerase II in the transcription of the targeted gene. The AKR superfamily is amongst those genes targeted by TR/RXR complex. In this study, the expression of genes namely *RAB3B*, *SLC2A1, AKRIC2* and *HP* affects the TR/RXR activation. There is growing evidence that some members of the AKR superfamily are induced during the development of chemoresistance in a variety of cancers (Deng et al., 2004). In colon cancers, studies reported that the up-regulated expression of *AKR1B10*, *AKR1C1*, *AKR1C2* and *AKR1C3* contributed to chemotherapeutic drug resistance indirectly, by reducing oxidative stress generated by these chemotherapeutic agents (Deng et al., 2004). Consequently, AKR1 enzyme inhibitors could be useful since they would be able to enhance the therapeutic efficacy of anticancer drugs by preventing chemoresistance (Shen et al., 2011).

The PXR/RXR heterodimer plays a fundamental role by regulating the expression of a critical set of protective gene products involved in xenobiotic and endobiotic metabolism. Studies reported that *GSTA1* and *SULT2A1* were amongst the genes identified as PXR/RXR target. Both genes were down-regulated in this study. Flavonoids namely quercetin and kaempferol were extensively reported to possess anti-cancer properties and regulates xenobiotic metabolism by activating the activity of nuclear receptors (Avior et al., 2013; Wang et al., 2014). Our previous study also showed a significant down-regulation of *CYP24A1* in HepG2 cells treated with *T. indica* leaf which contained a considerable amount of flavonoids including quercetin (Razali et al., 2015). Hence, quercetin detected in BLE might potentiate the activation of RXR heterodimers in BLE-treated HepG2 cells.

## Conclusion

BLE was found to affect the expression of many genes including those encoding proteins related to energy metabolism in cancer cells. It may increase the level of methylglyoxal via down-regulation of genes encoding methylglyoxal degradation enzymes. Additionally, it also up-regulated genes related to glycolytic metabolism which can further enhance the level of methylglyoxal. The chemopreventive potential of BLE could be further elucidated using different types of cancer cell lines, eventually moving to animal models. In the event where the cytotoxic effect of methylglyoxal is absent in other cells that rely on aerobic glycolysis for energy supply, BLE could therefore serve as a novel chemopreventive agent.

##  Supplemental Information

10.7717/peerj.2379/supp-1 Supplemental Information 1 Image for tcRNA integrity analysis (S1), List of significantly regulated genes (S2) **Sl:** Analysis of the integrity of the extracted tcRNA. The integrity of the tcRNA was evaluated using a denaturing gel electrophoresis. Two distinct bands that correlate with the ribosomal 28S and 18S were detected where the former is approximately twice than that of the latter. L1, L2, L3: BLE-treated samples; C1, C2, C3: control samples.BLE, leaf water extract of *B. racemosa*. **S2:** Genes significantly regulated by BLE, at 1.5 < fold change < − 1.5.

## Additional Information and Declarations

### Microarray Data Deposition

The following information was supplied regarding the deposition of microarray data: GSE79473ID: 200079473.

### Data Availability

The following information was supplied regarding data availability: The raw data has been supplied as a Supplemental File.
