# Supplementary material for: Antioxidant-rich leaf extract of Barringtonia racemosa significantly alters the in vitro expression of genes encoding enzymes that are involved in methylglyoxal degradation III
Source: PeerJ. 2016 Aug 25;4:e2379. doi: 10.7717/peerj.2379 (PMC5012310; doi:10.7717/peerj.2379)
Supplement: Supplemental Information 1 — Sl: Analysis of the integrity of the extracted tcRNA. The integrity of the tcRNA was evaluated using a denaturing gel electrophoresis. Two distinct bands that correlate with the ribosomal 28S and 18S were detected where the former is approximately twice than that of the latter. L1, L2, L3: BLE-treated samples; C1, C2, C3: control samples.BLE, leaf water extract of B. racemosa. S2: Genes significantly regulated by BLE, at 1.5 < fold change < − 1.5. [file peerj-04-2379-s001.doc]

**Supplementary Data**

**S1:** Primer sequences for selected genes used for validation of microarray data using real time-PCR

| Gene name | GenBank ID | Primer sequences | Size of PCR product (bp) |
| --- | --- | --- | --- |
| *GAPDH* | NM_002046 | Forward: 5’ GAAATCCCATCACCATCTTCCAGG 3’  Reverse: 5’ GAGCCCCAGCCTTCTCCATG 3’ | 120 |
| *ACTB* | NM_001101 | Forward: 5’ ACAGAGCCTCGCCTTTGCCG 3’  Reverse: 5’ ACATGCCGGAGCCGTTGTCG 3’ | 104 |
| *SERPINE1* | NM_000602 | Forward: 5’ AGGACGAACCGCCAATCGCA 3’  Reverse: 5’ GCCAGGTGGGCCACGTAGGAT 3’ | 146 |
| *IL8* | NM_000584 | Forward: 5’ CAGCCAAAACTCCACAGTCA 3’  Reverse: 5’ TTGGAGAGCACATAAAAACATCT 3’ | 155 |
| *AREG* | NM_001657 | Forward: 5’ CCCCAAGCCTTCGAGAGCGG 3’  Reverse: 5’ CGGTCTCTGGGGCAACTCGG 3’ | 109 |
| *EGR1* | NM_001964 | Forward: 5’ GACCGCAGAGTCTTTTCCTG 3’  Reverse: 5’ TGGGTTGGTCATGCTCACTA 3’ | 202 |
| *ITGA2* | NM_002203 | Forward: 5’ CGGGGGAGAGAAGCCCTCTGG 3’  Reverse: 5’ GGTCTGACCGGGGGACCGTA 3’ | 119 |
| *NT5E* | NM_002526 | Forward: 5’ CACCGCTACGGCCAGTCCAC 3’  Reverse: 5’ TGGGCACTCGACACTTGGTGC 3’ | 130 |
| *HAMP* | NM_021175 | Forward: 5’ ACAGCCAGACAGACGGCACG 3’  Reverse: 5’ TGCAGCTCTGCAAGTTGTCCCG 3’ | 133 |
| *GSTA1* | NM_145740 | Forward: 5’ AGTTTCTACAGCCTGGCAGCCC 3’  Reverse: 5’ AGTTCTTGGCCTCCATGACTGCG 3’ | 109 |

**S2:** Analysis of the integrity of the extracted tcRNA. The integrity of the tcRNA was evaluated using a denaturing gel electrophoresis. Two distinct bands that correlate with the ribosomal 28S and 18S were detected where the former is approximately twice than that of the latter. L1, L2, L3: BLE-treated samples; C1, C2, C3: control samples. BLE, leaf water extract of *B. racemosa*.

**S3:** Genes significantly regulated by BLE, at 1.5 < fold change < -1.5.

| No. | Genebank ID | Gene Symbol | Encoded Protein | Fold-Change |
| --- | --- | --- | --- | --- |
|  | NM_000602 | *SERPINE1* | Serpin peptidase inhibitor, clade E (nexin, plasminogen activator inhibitor type 1) | 2.8 |
|  | NM_000596 | *IGFBP1* | Insulin-like growth factor binding protein 1 | 2.4 |
|  | NM_001137550 | *LRRFIP1* | Leucine rich repeat (in FLII) interacting protein 1 | 2.3 |
|  | NM_001127345 | *GAGE12B* | G antigen 12B | 2.2 |
|  | NM_000584 | *IL8* | Interleukin 8 | 2.2 |
|  | NM_178012 | *TUBB2B* | Tubulin, beta 2B | 2.1 |
|  | NM_001657 | *AREG* | Amphiregulin | 2.0 |
|  | NM_014331 | *SLC7A11* | Solute carrier family 7, (cationic amino acid transporter light chain, y+L system) | 1.9 |
|  | NM_002867 | *RAB3B* | RAB3B, member RAS oncogene family | 1.9 |
|  | NM_024847 | *TMC7* | Transmembrane channel-like 7 | 1.9 |
|  | NM_005909 | *MAP1B* | Microtubule-associated protein 1B | 1.9 |
|  | NM_001964 | *EGR1* | Early growth response 1 | 1.8 |
|  | NM_005242 | *F2RL1* | Coagulation factor II (thrombin) receptor-like 1 | 1.8 |
|  | NM_003670 | *BHLHE40* | Basic helix-loop-helix family, member e40 | 1.8 |
|  | NM_002305 | *LGALS1* | Lectin, galactoside-binding, soluble, 1 | 1.8 |
|  | NM_005139 | *ANXA3* | Annexin A3 | 1.8 |
|  | NM_004331 | *BNIP3L* | Adenovirus E1B 19kDa interacting protein 3-like | 1.8 |
|  | NM_003383 | *VLDLR* | Very low density lipoprotein receptor | 1.8 |
|  | NM_005100 | *AKAP12* | A kinase (PRKA) anchor protein 12 | 1.7 |
|  | NM_005620 | *S100A11* | S100 calcium binding protein A11 | 1.7 |
|  | NM_002203 | *ITGA2* | Integrin, alpha 2 (CD49B, alpha 2 subunit of VLA-2 receptor) | 1.7 |
|  | NM_001031716 | *OBFC2A* | Oligonucleotide/oligosaccharide-binding fold containing 2A | 1.7 |
|  | NM_001127257 | *SLC39A10* | Solute carrier family 39 (zinc transporter), member 10 | 1.7 |
|  | NM_006472 | *TXNIP* | Thioredoxin interacting protein | 1.7 |
|  | NM_001443 | *FABP1* | Fatty acid binding protein 1, liver | 1.7 |
|  | NM_004696 | *SLC16A4* | Solute carrier family 16, member 4 (monocarboxylic acid transporters) | 1.7 |
|  | NM_006516 | *SLC2A1* | Solute carrier family 2 (facilitated glucose transporter), member 1 | 1.7 |
|  | NM_003243 | *TGFBR3* | Transforming growth factor, beta receptor III | 1.7 |
|  | NM_030666 | *SERPINB1* | Serpin peptidase inhibitor, clade B (ovalbumin), member 1 | 1.7 |
|  | NM_001712 | *CEACAM1* | Carcinoembryonic antigen-related cell adhesion molecule 1 (biliary glycoprotein) | 1.7 |
|  | NM_031213 | *FAM108A1* | Family with sequence similarity 108, member A1 | 1.7 |
|  | NM_005165 | *ALDOC* | Aldolase C, fructose-bisphosphate | 1.7 |
|  | NM_004751 | *GCNT3* | Glucosaminyl (N-acetyl) transferase 3, mucin type | 1.6 |
|  | NM_006216 | *SERPINE2* | Serpin peptidase inhibitor, clade E (nexin, plasminogen activator inhibitor type 1) | 1.6 |
|  | NM_032873 | *UBASH3B* | Ubiquitin associated and SH3 domain containing B | 1.6 |
|  | NM_003254 | *TIMP1* | TIMP metallopeptidase inhibitor 1 | 1.6 |
|  | NM_000096 | *CP* | Ceruloplasmin (ferroxidase) | 1.6 |
|  | NM_002276 | *KRT19* | Keratin 19 | 1.6 |
|  | NM_001069 | *TUBB2A* | Tubulin, beta 2A | 1.6 |
|  | NM_017784 | *OSBPL10* | Oxysterol binding protein-like 10 | 1.6 |
|  | NR_027354 | *LOC645166* | Lymphocyte-specific protein 1 pseudogene | 1.6 |
|  | NM_001343 | *DAB2* | Disabled homolog 2, mitogen-responsive phosphoprotein (Drosophila) | 1.5 |
|  | NM_001748 | *CAPN2* | Calpain 2, (m/II) large subunit | 1.5 |
|  | NM_001769 | *CD9* | CD9 molecule | 1.5 |
|  | NM_002526 | *NT5E* | 5'-nucleotidase, ecto (CD73) | 1.5 |
|  | NM_006931 | *SLC2A3* | Solute carrier family 2 (facilitated glucose transporter), member 3 | 1.5 |
|  | NM_000271 | *NPC1* | Niemann-Pick disease, type C1 | 1.5 |
|  | NM_001145199 | *C12orf75* | Chromosome 12 open reading frame 75 | 1.5 |
|  | NM_174894 | *TRAPPC5* | Trafficking protein particle complex 5 | -1.5 |
|  | NM_006845 | *KIF2C* | Kinesin family member 2C | -1.5 |
|  | NM_001801 | *CDO1* | Cysteine dioxygenase, type I | -1.5 |
|  | NM_001113738 | *ARL17A* | ADP-ribosylation factor-like 17A | -1.5 |
|  | NM_001976 | *ENO3* | Enolase 3 (beta, muscle) | -1.5 |
|  | NM_015169 | *RRS1* | RRS1 ribosome biogenesis regulator homolog (S. cerevisiae) | -1.5 |
|  | NM_032117 | *MND1* | Meiotic nuclear divisions 1 homolog (S. cerevisiae) | -1.5 |
|  | NM_139286 | *CDC26* | Cell division cycle 26 homolog (S. cerevisiae) | -1.5 |
|  | NM_002388 | *MCM3* | Minichromosome maintenance complex component 3 | -1.5 |
|  | NM_005915 | *MCM6* | Minichromosome maintenance complex component 6 | -1.5 |
|  | NM_014015 | *DEXI* | Dexi homolog (mouse) | -1.5 |
|  | NR_002437 | *SNORD54* | Small nucleolar RNA, C/D box 54 | -1.5 |
|  | NM_000386 | *BLMH* | Bleomycin hydrolase | -1.5 |
|  | NM_020394 | *ZNF695* | Zinc finger protein 695 | -1.5 |
|  | NM_016567 | *BCCIP* | BRCA2 and CDKN1A interacting protein | -1.5 |
|  | NM_152551 | *SNRNP48* | Small nuclear ribonucleoprotein 48kDa (U11/U12) | -1.5 |
|  | NR_003925 | *RNU4-1* | RNA, U4 small nuclear 1 | -1.5 |
|  | NM_015465 | *GEMIN5* | Gem (nuclear organelle) associated protein 5 | -1.5 |
|  | NM_003095 | *SNRPF* | Small nuclear ribonucleoprotein polypeptide F | -1.5 |
|  | NR_002973 | *SNORA40* | Small nucleolar RNA, H/ACA box 40 | -1.5 |
|  | NM_014673 | *TTC35* | Tetratricopeptide repeat domain 35 | -1.5 |
|  | NM_016625 | *RSRC1* | Arginine/serine-rich coiled-coil 1 | -1.5 |
|  | NM_003000 | *SDHB* | Succinate dehydrogenase complex, subunit B, iron sulfur (Ip) | -1.5 |
|  | NM_175617 | *MT1E* | Metallothionein 1E | -1.5 |
|  | NM_017785 | *CCDC99* | Coiled-coil domain containing 99 | -1.6 |
|  | NM_004578 | *RAB4A* | RAB4A, member RAS oncogene family | -1.6 |
|  | NM_018077 | *RBM28* | RNA binding motif protein 28 | -1.6 |
|  | NM_006347 | *PPIH* | Peptidylprolyl isomerase H (cyclophilin H) | -1.6 |
|  | NM_001025195 | *CES1* | Carboxylesterase 1 | -1.6 |
|  | NM_032315 | *SLC25A33* | Solute carrier family 25, member 33 | -1.6 |
|  | NR_000007 | *SNORD73A* | Small nucleolar RNA, C/D box 73A | -1.6 |
|  | NM_024094 | *DSCC1* | Defective in sister chromatid cohesion 1 homolog (S. cerevisiae) | -1.6 |
|  | NM_024307 | *GDPD3* | Glycerophosphodiester phosphodiesterase domain containing 3 | -1.6 |
|  | NM_001040431 | *CCDC56* | Coiled-coil domain containing 56 | -1.6 |
|  | NM_005030 | *PLK1* | Polo-like kinase 1 | -1.6 |
|  | NM_020242 | *KIF15* | Kinesin family member 15 | -1.6 |
|  | NM_022746 | *MOSC1* | MOCO sulphurase C-terminal domain containing 1 | -1.6 |
|  | NM_006938 | *SNRPD1* | Small nuclear ribonucleoprotein D1 polypeptide 16kDa | -1.6 |
|  | NM_001054 | *SULT1A2* | Sulfotransferase family, cytosolic, 1A, phenol-preferring, memb | -1.6 |
|  | NM_032359 | *C3orf26* | Chromosome 3 open reading frame 26 | -1.6 |
|  | NM_030980 | *ISG20L2* | Interferon stimulated exonuclease gene 20kDa-like 2 | -1.6 |
|  | NM_001080443 | *KIF18B* | Kinesin family member 18B | -1.6 |
|  | NM_005733 | *KIF20A* | Kinesin family member 20A | -1.6 |
|  | NR_002560 | *SNORD31* | Small nucleolar RNA, C/D box 31 | -1.6 |
|  | NM_005143 | *HP* | Haptoglobin | -1.6 |
|  | NM_001012410 | *SGOL1* | Shugoshin-like 1 (S. pombe) | -1.6 |
|  | NM_030906 | *STK33* | Serine/threonine kinase 33 | -1.6 |
|  | NM_017742 | *ZCCHC2* | Zinc finger, CCHC domain containing 2 | -1.6 |
|  | NM_015918 | *POP5* | Processing of precursor 5, ribonuclease P/MRP subunit (S. cerevisiae) | -1.6 |
|  | NR_022008 | *PAR5* | Prader-Willi/Angelman syndrome-5 | -1.6 |
|  | NM_032111 | *MRPL14* | Mitochondrial ribosomal protein L14 | -1.6 |
|  | NM_017858 | *TIPIN* | TIMELESS interacting protein | -1.6 |
|  | NM_005952 | *MT1X* | Metallothionein 1X | -1.6 |
|  | NM_022451 | *NOC3L* | Nucleolar complex associated 3 homolog (S. cerevisiae) | -1.6 |
|  | NM_006461 | *SPAG5* | Sperm associated antigen 5 | -1.6 |
|  | NM_019042 | *PUS7* | Pseudouridylate synthase 7 homolog (S. cerevisiae) | -1.6 |
|  | NR_003942 | *SNORD76* | Small nucleolar RNA, C/D box 76 | -1.6 |
|  | NM_016391 | *NOP16* | NOP16 nucleolar protein homolog (yeast) | -1.7 |
|  | NR_002743 | *SNORD50A* | Small nucleolar RNA, C/D box 50A | -1.7 |
|  | NM_017769 | *G2E3* | G2/M-phase specific E3 ubiquitin protein ligase | -1.7 |
|  | NM_032338 | *LLPH* | LLP homolog, long-term synaptic facilitation (Aplysia) | -1.7 |
|  | NM_001011663 | *PCGF6* | Polycomb group ring finger 6 | -1.7 |
|  | NM_020995 | *HPR* | Haptoglobin-related protein | -1.7 |
|  | NM_020861 | *ZBTB2* | Zinc finger and BTB domain containing 2 | -1.7 |
|  | NM_001013742 | *DGKK* | Diacylglycerol kinase, kappa | -1.7 |
|  | NM_003524 | *HIST1H2BH* | Histone cluster 1, H2bh | -1.7 |
|  | NM_001142930 | *API5* | Apoptosis inhibitor 5 | -1.8 |
|  | NM_018264 | *TYW1* | tRNA-yW synthesizing protein 1 homolog (S. cerevisiae) | -1.8 |
|  | NM_178493 | *NOTUM* | Notum pectinacetylesterase homolog (Drosophila) | -1.8 |
|  | NM_005980 | *S100P* | S100 calcium binding protein P | -1.8 |
|  | NM_019083 | *CCDC76* | Coiled-coil domain containing 76 | -1.8 |
|  | NM_153608 | *ZNF114* | Zinc finger protein 114 | -1.8 |
|  | NM_018344 | *SLC29A3* | Solute carrier family 29 (nucleoside transporters), member 3 | -1.8 |
|  | NR_015440 | *FLJ42875* | Hypothetical LOC440556 | -1.9 |
|  | NM_001146108 | *PTGR1* | Prostaglandin reductase 1 | -1.9 |
|  | NM_016183 | *MRTO4* | mRNA turnover 4 homolog (S. cerevisiae) | -1.9 |
|  | NM_005123 | *NR1H4* | Nuclear receptor subfamily 1, group H, member 4 | -1.9 |
|  | NM_005950 | *MT1G* | Metallothionein 1G | -2.0 |
|  | NM_003167 | *SULT2A1* | Sulfotransferase family, cytosolic, 2A, dehydroepiandrosterone | -2.0 |
|  | NM_000670 | *ADH4* | Alcohol dehydrogenase 4 (class II), pi polypeptide | -2.1 |
|  | NM_001354 | *AKR1C2* | Aldo-keto reductase family 1, member C2 (dihydrodiol dehydrogenase) | -2.1 |
|  | NM_002167 | *ID3* | Inhibitor of DNA binding 3, dominant negative helix-loop-helix protein | -2.1 |
|  | NM_020299 | *AKR1B10* | Aldo-keto reductase family 1, member B10 (aldose reductase) | -2.2 |
|  | NM_005949 | *MT1F* | Metallothionein 1F | -2.2 |
|  | NM_181353 | *ID1* | Inhibitor of DNA binding 1, dominant negative helix-loop-helix protein | -2.3 |
|  | NM_133367 | *PAQR8* | Progestin and adipoQ receptor family member VIII | -2.3 |
|  | NM_000782 | *CYP24A1* | Cytochrome P450, family 24, subfamily A, polypeptide 1 | -2.4 |
|  | NM_002166 | *ID2* | Inhibitor of DNA binding 2, dominant negative helix-loop-helix protein | -2.7 |
|  | NM_145740 | *GSTA1* | Glutathione S-transferase alpha 1 | -6.1 |
|  | NM_021175 | *HAMP* | Hepcidin antimicrobial peptide | -6.5 |

**S4:** IPA analyses of the top networks associated to the significantly altered expression genes in HepG2 cells in response to BLE treatment

| Top network | Score | Molecules in network |
| --- | --- | --- |
| 1. Cancer, Cell Death and Survival, Cellular Movement | 44 | AKAP12, AKR1B10, Ap1, AREG/AREGB, BHLHE40, CAPN2, CD9, CEACAM1, Cyclin A, CYP24A1, DAB2, ERK1/2,F2RL1, Focal adhesion kinase, GOT, Hdac, Histone h4, ID1, ID3, IGFBP1, IL8, ITGA2, KRT19 (includes EG:16669), LDL, MCM3, Mek, MT1E, MT1F, Notch, NPC1, PDGF BB, PUS7, S100A11, SLC2A3, TXNIP |
| 1. Cancer, Cardiac Dilation, Cardiovascular System Development and Function | 32 | Akt, Alpha catenin, Cg, EGR1, ENO3, ERK, estrogen receptor, FABP1, GSTA1, Histone h3, Ige, IgG, Igm, Jnk, KIF2C, LGALS1, MAP1B, MT1G, NFkB (complex), NR1H4, NT5E, P38 MAPK, PI3K (complex), Pkc(s), PLK1, RNA polymerase II, SERPINE1, SLC2A1, SULT2A1, TCR, TGFBR3, TIMP1, TIPIN, TUBB2A, UBASH3B |
| 1. Cell Cycle, Connective Tissue Development and Function, Cellular Development | 27 | AICDA, AKR1C1/AKR1C2, ANXA3, CCNE1, CEBPA, DHCR7, E2F4, G0S2, GTF2B, HIST1H2BH/HIST1H2BO, HPR, Hsp90, ID2, ID3, Ifi204 (includes others), IGF1R, IGHE, IRGM, KIF20A, MCM4, MCM6, miR-99a-5p (and other miRNAs w/seed ACCCGUA), MRTO4, NOC3L, PLK1, PPARA, PRC1 (includes EG:233406), PRDM5, PTGR1, RNU4-1, SNRNP48, SPAG5, SULT2A1, VLDLR, XDH |
| 1. Metabolic Disease, Drug Metabolism, Endocrine System Development and Function | 25 | ADH4 (includes EG:127), ALDOC, BHLHE40, BMP4, BMP6, BNIP3L, CACNA1B, CD74, CDO1, FKBP5, HAMP, HDAC6, HFE2, HIF1A, IGF1, LOC645166, NR3C1, PDGFA, POR, RAB3B, S100P, SDHB, SIAH2, SLC16A4, SLC2A1, SLC39A10, SLC40A1, SRD5A1, TGM2 (includes EG:21817), TMPRSS6, TNFRSF12A, TRMT13, TUBB2A, TUBB2B, ZBTB20 |
| 1. Behaviour, Cell-To-Cell Signalling and Interaction, Drug Metabolism | 21 | ACTG2, ADCYAP1 (includes EG:11516), AKAP12, BNIP3L, CCDC99, CD163, DGKK, Eotaxin, FKBP5, FSH, GAD1 (includes EG:100006588), GDPD3, GPR56, HP, IL1B, KIF15, Lh, MAPK1, MT1X, NPC2 (includes EG:10577), NT5E, Orm1 (includes others), PLCL1,POP5 (includes EG:117109), PPIH, RAB4A, SERPINB1, SGOL1, SPN, STK17A, STX4, TLN1, TNFAIP6, TOB1, VEGFC |
